# To fund or not to fund?

**DOI:** 10.7554/eLife.32015

**Published:** 2017-09-28

**Authors:** Sarah Shailes

**Affiliations:** eLifeCambridgeUnited Kingdom

**Keywords:** peer review, funding agencies, bias, science policy

## Abstract

Funding agencies use many different criteria and peer review strategies to assess grant proposals.

Earlier this year, the European Research Council (ERC) marked its tenth anniversary with a week of events in various countries. These events celebrated the fact that, among other things, the council has awarded substantial grants to over 7,000 scientists, the majority of whom are under 40 years old, and that ERC grants have contributed to at least 100,000 research papers. However, these 7,000 were the lucky few – last year the council received a total of 7,500 applications and funded just 12% of them.

The ERC is not alone in being highly selective; the National Institutes of Health in the US, the Biotechnology and Biological Sciences Research Council in the UK, and the Medical Research Council, also in the UK, have all reported success rates of less than 30% for grant proposals (Matthews, 2016). In almost all cases the decision to fund or not to fund a proposal will have been taken after two or more of the applicant's peers have assessed the application (or an outline or summary of the application).

## Criteria, criteria, criteria

The sole focus at the ERC is on scientific excellence. "The aim is to recognize the best ideas, and confer status and visibility on the best brains in Europe, while also attracting talent from abroad," says a spokesperson for the council. Some critics have claimed that this strategy unfairly favors researchers based at institutions in wealthier nations, with two-thirds of ERC grants going to researchers based in just five countries – the UK, Germany, France, the Netherlands and Switzerland – between 2007 and 2013. However, the ERC spokesperson disputes this: "The peer review evaluation process has been carefully designed to identify scientific excellence irrespective of the gender, age, nationality or institution of the principal investigator and other potential biases." The ERC also takes career breaks and "unconventional career paths" into account when awarding grants.

Most other funding agencies use two or more criteria to assess research proposals. The National Science Foundation (NSF) – the primary source of federal funding for non-medical basic research in the US – assesses all applications on two major criteria: intellectual merit and broader impacts. "The broader impacts criterion encompasses the potential to benefit society and contribute to the achievement of specific, desired societal outcomes," says Rob Margetta, a public affairs specialist at the NSF.

Cancer Research UK – the world's largest cancer research charity – assesses the expertise of the research team submitting the proposal and the originality of the ideas along with several other criteria including the relevance to the charity's current research priorities, the quality of the experimental design, and value for money. "The criteria are outlined on our website," says Matt Kaiser, head of discovery research at the charity, "so that applicants really understand what they will be judged on."

At the Australian Research Council (ARC), different funding schemes employ different criteria to assess applications. For example, in a scheme that supports early career researchers, the investigator themselves and "project quality and innovation" are the two major criteria, accounting for 75% of the assessment score. However, in schemes that promote collaborations with industry and other partners, emphasis is placed on the commitment from partner organizations and project significance and innovation, with the investigators criteria carrying less weight.

Most funding agencies use two or more criteria to assess research proposals.

The ARC also tries to avoid various forms of bias when making decisions. "Researchers who are early in their research career or have had an interrupted research career – including employment outside academia, unemployment, child birth, carers' responsibilities and other personal circumstances – will have this taken into account," says Leanne Harvey, who is the council's executive general manager.

## Ask the experts

Despite using different sets of criteria for different funding schemes, all proposals for ARC funding pass through the same peer review process. Firstly, two or more Detailed Assessors – researchers who have expertise in the same field as the proposal – provide written assessments and ratings based on the relevant criteria. The applicants are then given the opportunity to provide feedback on these assessments before the application is passed on to two or more General Assessors, who are researchers working in the same or related fields. The General Assessors use their own expertise, plus their broader knowledge of research planning and other issues, to examine the written assessments and applicant feedback and give each application an initial assessment score. The initial scores are used to produce a ranked list of proposals for a committee of General Assessors to discuss. This committee makes recommendations that are considered by the CEO and then passed on to the Minister, who determines which proposals should be funded.

Virtually all applications submitted to the NSF, which received a staggering 49,620 research proposals in the 2015 fiscal year alone, are independently evaluated by at least three external experts who provide written assessments. An NSF program officer examines these assessments and makes recommendations for funding. Before an application can be funded it must be signed off by the relevant division director and then reviewed for business, financial and policy implications by another NSF official.

Other funding agencies, including Cancer Research UK (CRUK) and the ERC, often use two-step peer review processes to reduce the burden on external reviewers. A panel of external experts who work in the relevant research fields initially screens the proposals based on an outline or summary. Only the proposals that pass this first stage are sent to two or more independent experts, who are asked to provide detailed reviews on the full applications. The original panel then examines these reviews and is responsible for making the final decision about whether to fund the research. For large grant applications to CRUK, the panel may also interview the applicant, providing him or her with the opportunity to discuss and counter any concerns.

"For most grants, and depending on the size of the award, we seek anywhere between three and six written reviews," says Kaiser of CRUK. The number of reviewers is an important consideration because two people assessing the same research proposal can come to different conclusions, making it difficult for decision makers to score the proposal and rank it against others. In 2012, a study that examined data on peer review at the Fonds zur Förderung der wissenschaftlichen Forschung in Austria recommended that bioscience research proposals should be assessed by a minimum of three independent reviewers to help compensate for this variation (Mutz et al., 2012).

Once a funding agency has recruited reviewers, it needs to ensure that they follow the agency's policies and apply its selection criteria correctly. At the ERC this starts with the President of the council briefing the chairs of its 25 peer review panels on the key principles of the ERC evaluation process, including its policy on gender balance, measures on widening participation, conflicts of interest and matters of confidentiality.

As a member of the Association of Medical Research Charities, CRUK is bound by the association's principles of peer review. "This means our review processes are independent, impartial, balanced, accountable and include a diversity of opinion," says Kaiser. To ensure CRUK is meeting these requirements, the charity's review processes are audited every five years.

## A different approach

The Volkswagen Foundation – a private research agency founded in 1962 in Germany – has been testing out alternative ways to assess applications for some of its funding schemes. For example, in the *Experiment!* scheme, which supports researchers in science and engineering who want to investigate daring new ideas, double-blind peer review ensures that the interdisciplinary jury reviewing the applications assesses the quality of the idea and not the reputation of the person behind it.

Since *Experiment!* grants are so broad in scope it is possible that funding decisions could be biased towards fields that the jury are more familiar with. To address this issue, the Volkswagen Foundation is currently running a trial that uses a lottery to increase the diversity of the proposals they fund. First, the foundation screens the applications to make sure that they address the program criteria. Anonymized versions of the shortlisted applications are then passed to the jury, which rejects any that are not of sufficient quality. From the pool of good quality proposals that remains, the jury selects the ideas they find most convincing to receive funding. The other good quality proposals have a second chance to be awarded funding through the lottery overseen by the foundation's legal officer. In this way the foundation hopes to "uncover projects which otherwise are easily overlooked," says program director Ulrike Bischler. "Depending on the results of an independent evaluation, this partial randomization approach might be used in more funding initiatives in the future."

Of course the story does not end here. Once a researcher has received his or her grant they will need to do the research that they promised to do. If all goes well, they will submit the results to a journal – where, once again, they will find themselves under scrutiny from two or more of their peers.

## Note

This Feature Article is part of a collection of articles on peer review.

## References

[bib1] Matthews D (2016). UK grant success rates prompt worldwide comparisons. Times Higher Education.

[bib2] Mutz R, Bornmann L, Daniel HD (2012). Heterogeneity of inter-rater reliabilities of grant peer reviews and its determinants: a general estimating equations approach. PLoS One.

